# Omission of Chemotherapy in Early Stage Nasopharyngeal Carcinoma Treated with IMRT: A Paired Cohort Study

**DOI:** 10.1097/MD.0000000000001457

**Published:** 2015-10-02

**Authors:** Tingting Xu, Chunying Shen, Guopei Zhu, Chaosu Hu

**Affiliations:** From the Department of radiation oncology, Fudan University Shanghai Cancer Center, Shanghai, China.

## Abstract

The objective of this study was to evaluate the necessity of concurrent chemotherapy in T1-2N1 nasopharyngeal carcinoma (NPC) patients treated with intensity-modulated radiation therapy (IMRT).

The retrospective analysis was conducted using the paired comparison method. We matched cases to controls using the greedy matching algorithm with 1:1 control to case ratio. Controls were matched to cases by factors including age, gender, T stage, and duration of RT. The control group included patients received IMRT alone. In another group, concurrent chemotherapy (DDP 40 mg/m^2^/w) was administrated to each paired patient.

From Jan 2009 to Dec 2011, a total of 86 well-balanced T1-2N1 (2002 UICC staging system) NPC patients were retrospectively analyzed. Half of them (43 patients) received radical IMRT alone and another 43 received concurrent chemotherapy with IMRT (CCRT). Median follow-up is 37.4 months (4.8–66.2 months). All patients received a radiation dose of 66Gy/30Fx. In the CCRT group, all patients received a cumulative dose of ≥200 mg/m^2^. The differences of 3-year overall survival (OS), 3-year progression-free survival (PFS), 3-year relapse-free survival (RFS), and 3-year metastasis-free survival (MFS) between 2 groups were not significant (*P* > 0.05). The most frequently increased toxicities related to chemotherapy were mild to moderate leukopenia (*P* = 0.003) and mild anemia (*P* = 0.008).

Omission of weekly cisplatin chemotherapy resulted in comparable survival outcomes to CCRT in IMRT populations. More data from future randomized trials are warranted to further confirm it.

## INTRODUCTION

Nasopharyngeal carcinoma (NPC) is one of the most frequent malignant tumors in head and neck cancers, especially in southern China. Thanks to advances that had recently made in the strategies of basic education and popularizing regular physical examinations, more patients present as early stages. Early stage NPC has been considered one of the most curable malignant disease, ∼ 80% patients can be rendered disease-free in the long term even using the conventional technique.^1,2^

In two-dimensional conventional radiotherapy (2D-CRT) era, RT alone is recommended for stage I, whereas concurrent chemoradiotherapy (CCRT) is more acceptable for stage II disease. A phase III randomized study demonstrated adding chemotherapy statistically significantly improved the survival rate with more acute toxic effects in stage II NPC patients.^3^ Previously, our experience^4^ had also showed CCRT improved the 5-year RFS rate for T2N1M0 patients (91.5% vs 77.3%, *P* = 0.008). CCRT now is being recommended to the patients with stage II disease in the National Comprehensive Cancer Network (NCCN) guideline.^5^

However, with the development of intensity-modulated radiation therapy (IMRT), the treatment outcome has largely improved. Clinical research from many large, prospective clinical trials showed that the IMRT had led to impressively better long-term disease control and overall survival (OS) with less radiotherapy-related complications when compared to the 2D plan,^6–13^ especially in early T-stage disease.^14^ It is perhaps because of such dramatic advances of local control by IMRT that some investigators have been asking whether the same results could be obtained with less intensive approaches, one possible resolvent is to omit chemotherapy. Some had reported promising outcomes in NPC patients treated with IMRT without chemotherapy.^15–16^

Till recently, studies were unable to provide definite answers regarding this controversial issue. To answer this question, a single institution retrospectively analysis of patients treated with radical IMRT was conducted at Fudan University Shanghai Cancer Center. Another matched group treated in the same center with IMRT and concomitant weekly cisplatin chemotherapy was constituted for comparison.

## METERIALS AND METHODS

### Patient Eligibility

All patients diagnosed as stage T1-2N1M0 according to 2002 Union for International Cancer Control (UICC) staging system at Fudan University Shanghai Cancer Center between Jan 2009 and Dec 2011 were identified. The treatment decision in favor or against CRT in the individual patient was made according to the radiation oncologists’ clinical experience. Patients received < 5 cycles of cisplatin (200 mg/m^2^) were not included, other inclusion criteria were as follows: histologically confirmed NPC by biopsy, no evidence of distant metastasis, no previous treatment for NPC, no other concomitant malignant disease, using contrasted magnetic resonance imaging (MRI) as the staging method, and receiving radical IMRT at initial diagnosis.

A control group for comparison of the survival rate was identified from patients who received IMRT alone. As the observation group, patients were selected to match the age, gender, T stage, and duration of RT with a 1:1 ratio. For both groups, patients who received neoadjuvant chemotherapy and/or adjuvant chemotherapy were excluded. A paired observational design was used.

Routine workup included a thorough complete history, physical examination, complete blood cell count, and biochemical profile analysis, which were also conducted once every week during the radiotherapy/radiochemotherapy. Contrasted MRI of the nasopharynx and neck, chest computed tomography (CT), ultrasound of the abdomen, and bone scan were used for staging the disease at baseline. All patients provided their written informed consent before treatment and the study was approved by the Institutional Review Boards of Fudan University Shanghai Cancer Center.

### Treatment and Toxicity Assessments

The IMRT treatment plans were designed and optimized using an inverse planning system (Pinnacle 3, Philips).

The gross tumor volume (GTV) was contoured on the CT images including the visible lesions on the contrasted-MRI scans. The clinical tumor volume (CTV) represents the primary tumor with potential sub-clinical disease. Uniform planning margins (5 mm) were added to account for organ motion and setup uncertainty. The prescription doses to GTVnx (GTV of nasopharynx), GTVnd (GTV of lymph node), CTV1 (high-risk drainage region), and CTV2 (low risk drainage region) were 66Gy/30fx, 66Gy/30fx, 60Gy/30fx and 54Gy/30fx, respectively. Residuals of the primary lesions observed on the MRI scans at the end of treatment were treated with a local dose boost. The residual of the primary lesions were boosted using brachytherapy (192Irγ-ray) or x-ray by re-contouring the P-GTVnx-boost area on the CT simulation localization slices. Residual lymph nodes were boosted using x-ray by re-contouring the LN-boost area or electron beam.

Cisplatin was delivered weekly at a dose of 40 mg/m^2^ concurrently with radiotherapy in the CCRT group.

The response rate was evaluated using the Response Evaluation Criteria in Solid Tumors (RECIST). Acute toxicities were defined as occurring within 90 days of treatment completion using the Common Terminology Criteria for Adverse Events (CTCAE) v3.0. Late toxicities were evaluated according to the toxicity criteria of the Radiation Therapy Oncology Group (RTOG) at each follow-up.

### Follow up

After the completion of RT, patients were subsequently followed up every 3 months through the first 2 years, every 6 months for the next 3 years, and then annually. Physical examination, MRI of the nasopharynx and neck and ultrasound imaging of the abdomen were performed. Chest CT was performed annually, and bone scan was required if clinically indicated.

### Endpoints and Statistical Analysis

We matched cases to controls using the greedy matching algorithm with 1:1 control to case ratio which was performed by using SAS 9.2 (SAS Institute, Inc, Cary, NC). Controls were matched to cases by factors including age, gender, T stage, and duration of RT.

The primary end point of the study was the overall survival (OS). In the analysis of OS, a patient was considered to have death if he dead as a result of any cause. Other outcomes of interest included progression-free survival (PFS), locoregional relapse-free survival (RFS), metastasis-free survival (MFS), and the toxicities associated with the treatment. PFS was defined as the time from start of the RT to relapsed/metastasized. RFS and MFS were defined as the duration from the date of start of the RT to relapse or distant metastasis. Survival curves were estimated with the Kaplan–Meier method using the log-rank test (SPSS v20.0). The χ^2^ test was used to detect statistical differences in proportions. These statistical analyses were carried out at a 5% level of significance.

## RESULTS

### Patient and Tumor Characteristics

Finally, a total of 86 well-balanced patients (43 pairs) were retrospectively analyzed, of which 63 were men and 23 were women, with a sex ratio of 2.7:1. Median age was 50 years (29–73 years). There were 34, 14, and 38 patients with stage T1N1, T2aN1, and T2bN1, respectively, as shown in Table 1.

**TABLE 1:**
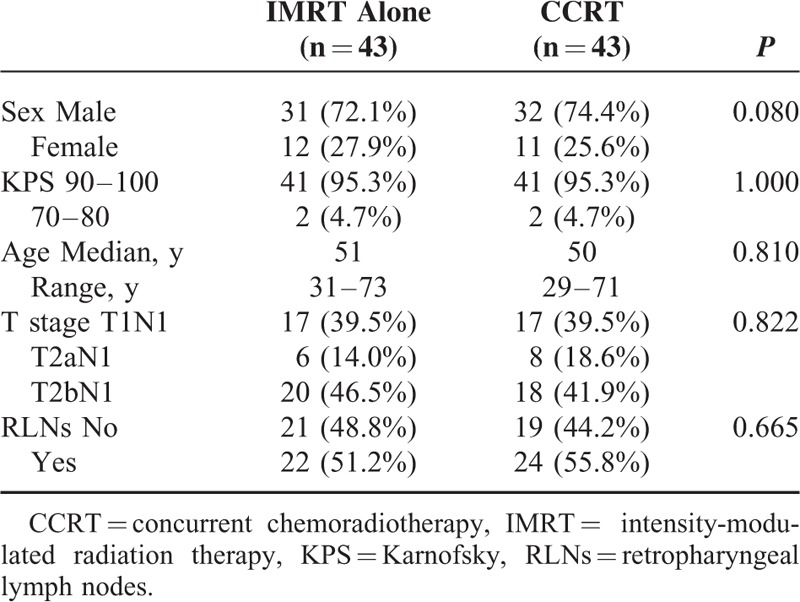
Patient Characteristics

### Treatment Compliance

All patients received a radiation dose of 66Gy/30Fx. The median duration time of RT was 43 days with a range of 40 to 52 days in the observation group and 44 days with a range of 40 to 50 days in the control group (*P* = 0.817) (Table 2). The same dose prescription of boosts was delivered to both groups. Boost to the nasopharynx was 4.4Gy/2Fx and 16Gy/2Fx by using x-ray and brachytherapy, respectively. Boost to the neck was 4.4Gy/2Fx and 4Gy/2Fx by using x-ray and electron beam, respectively.

**TABLE 2:**
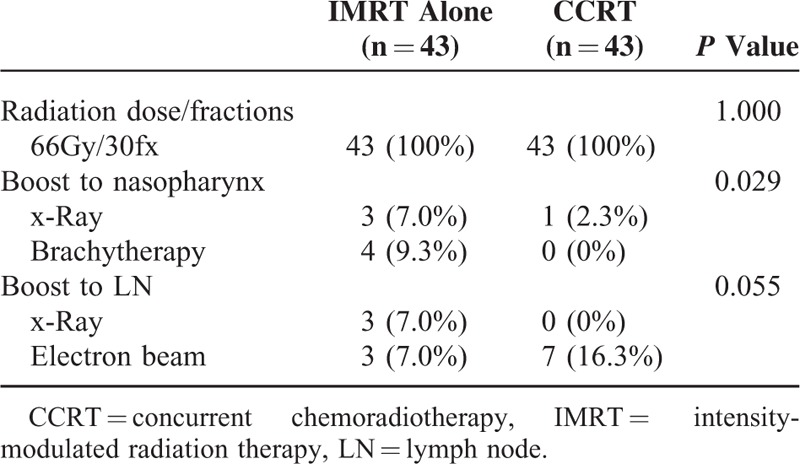
Radiotherapy Compliance

During CCRT, the percentage of patients receiving 6 and 5 infusions of weekly cisplatin was 16.3% and 83.7%, respectively.

### Response Rate

All populations achieved an overall response rate (CR+PR) of 100% (Table 3). The CR rates in the investigational and control arms were 72.1%, for both, at the end of RT. More patients had residual diseases of neck in the CCRT arm and more patients had residual diseases of nasopharynx in the IMRT alone arm (*P* = 0.028). Most of them received boosts to the residual locations. There were only 2 patients in each group who had residual neck lymph nodes 3 months after RT completion and received subsequently salvage neck dissections.

**TABLE 3:**
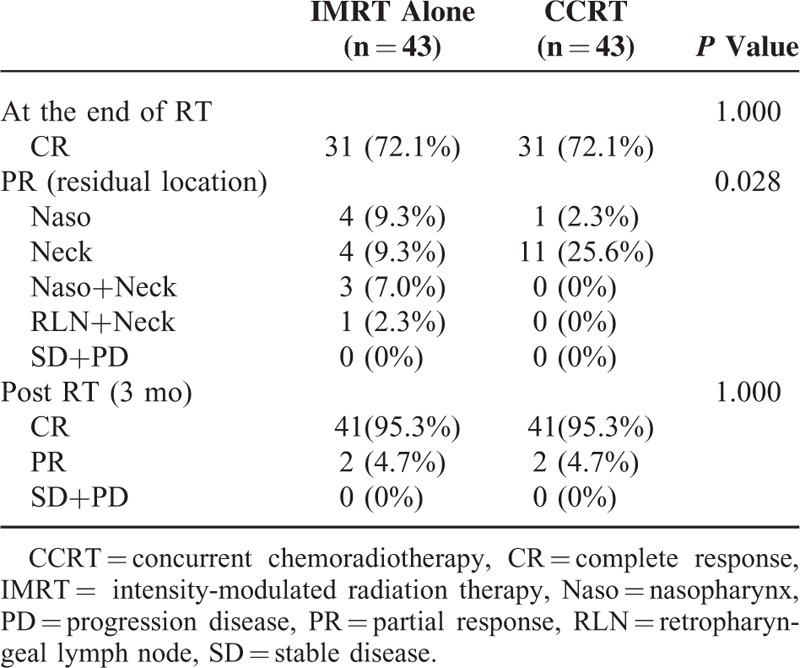
Response Rate

### Survival

**FIGURE 1:**
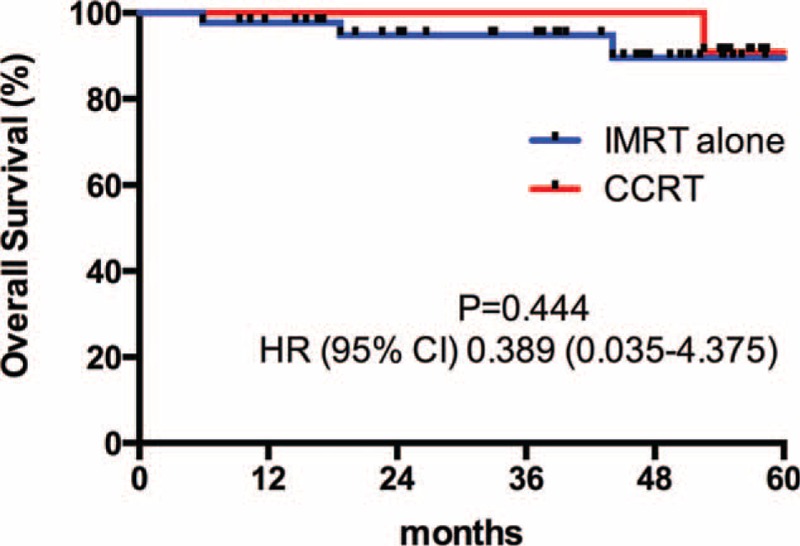
3-Year overall survival curve.

**FIGURE 2:**
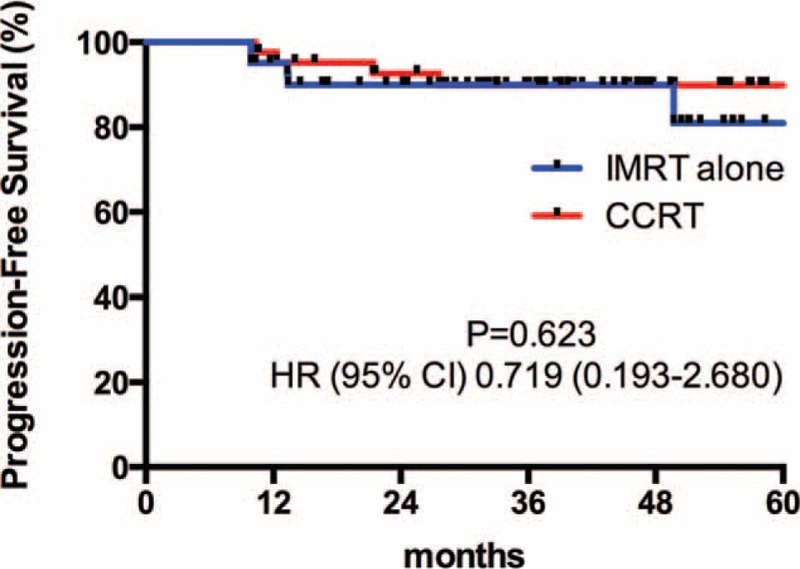
3-Year progression-free survival curve.

**FIGURE 3:**
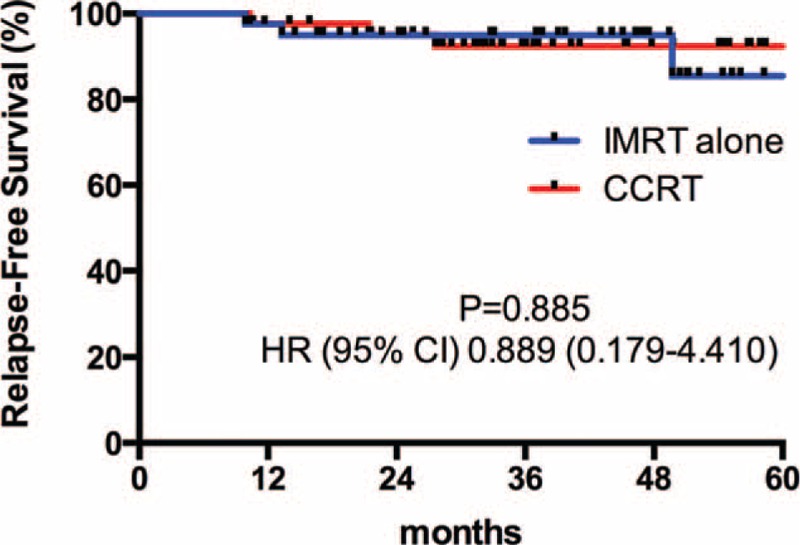
3-Year relapse-free survival curve.

**FIGURE 4:**
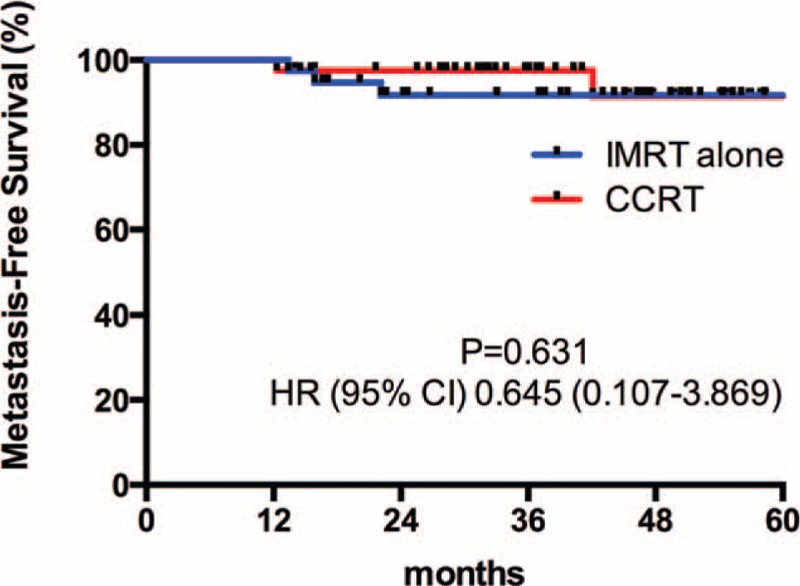
3-Year metastasis-free survival curve.

Patients were followed-up until death or a median of 37.4 months (range 4.8–66.2 months) among living patients. Four patients died before the time of the current analysis. Among them, 3 died of tumor progressions (2 from the IMRT alone group, 1 from the CCRT group), 1 died of unknown reason (from the IMRT alone group). The differences of 3-year OS (Figure 1), 3-year PFS (Figure 2), 3-year RFS (Figure 3), and 3-year MFS (Figure 4) between arms were not significant (Table 4).

**TABLE 4:**
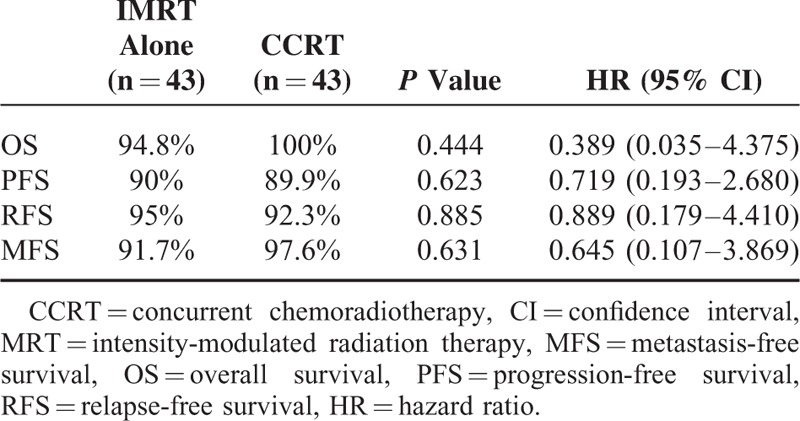
3-Year Survival Rate and Hazard Ratio of 2 Groups

### Toxicity

Acute toxicities during the treatment were listed in Table 5. The most frequent toxicities related to chemotherapy were mild to moderate leukopenia (*P* = 0.003) and mild anemia (*P* = 0.008). Intervenous nutritional support days were 2.09 ± 2.98 days (in 16 patients) and 2.74 ± 4.95 days (in 15 patients) in IMRT alone and CCRT group, respectively (*P* = 0.252).

**TABLE 5:**
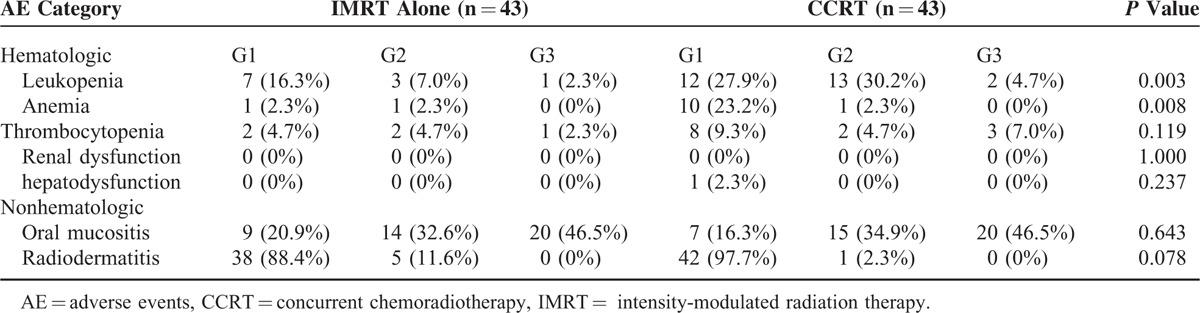
Highest Grades of Acute Adverse Events

Radiation-induced temporal lobe injuries were discovered in 1 patient (IMRT alone group, 34.5 months after RT completion) characterized by severe headache/memory change and confirmed by the contrasted-MRI scan. Other severe late adverse events included 3 severe trismus and 4 radiotherapy-induced caries. Three patients (1 from the IMRT alone group, 2 from the CCRT group) developed secondary primary tumors (lung, rectum, and thyroid).

## DISCUSSION

The aim of the current retrospectively paired study was to establish whether chemotherapy could be omitted from CCRT in N-positive stage IIB NPC patients treated with IMRT.

According to the 2002 UICC staging system, N1 is widely accepted as the independent prognostic factor that influences the distant control of early stage (stages I, II). Early stage patients with positive lymph nodes will have more chances to develop distant metastasis and obtain poor survival,^17,18^ whereas outcomes of another prognostic factor-T2b (parapharyngeal extension) were different from several studies. Xiao et al^19^ reported the 5-year MFS rate in the patients with the presence of negative and positive parapharyngeal space involvement were 88.1% and 73.8% (*P* < 0.05), respectively. Cheng et al^20^ further discovered that the metastatic risk of T2bN1 patients were significantly higher than T1–2aN1 patients (*P* = 0.03, HR 11.3 [1.3–102]). Notwithstanding, in Ng et al's study,^21^ no statistical significance was noted when using the MRI as the staging method and 3-dimensional (3D) conformal as the radiotherapy technique. The 5-year MFS and OS rates were pretty much the same thing, which was 87% and 83%, respectively for stage T2a and 91% and 86%, respectively for stage T2b (*P* > 0.05). However, recently, Tang et al^22^ demonstrated parapharyngeal extension remains a poor factor for MFS, especially concomitantly presented with positive lymph nodes in patients treated with IMRT (79.3% vs 92.0% *P* < 0.001). With the uncertainty of the impact of parapharyngeal extension on survival outcome, we only chose N1 as the adverse factor to avoid the possible bias. Additionally, Chua et al^2^ reported 10-year survival outcomes of early stage patients (stages I, II) and discovered T1–T2N1 disease had a relatively worse outcome (*P* < 0.001). They suggested more aggressive therapy for those patients. Therefore, we collected T1–2N1 patients who received the modern IMRT technique in our center to evaluate the exact value of concurrent chemotherapy in this population.

In view of the excellent tumor control (5-year LRFS: > 95%; 5-year DMFS:>95%) and survival rate (5-year OS: >85%) reported for early stage NPC using IMRT alone,^16,18^ a substantial proportion of patients who received CCRT are likely to be overtreated.

In the 2D-CRT era, improvement of OS by CCRT compared to RT alone was often derived from the increased local and/or distant control.^3,4^ Almost all confirmed therapeutic advantage of CCRT over RT alone was based on conventional radiation techniques. With the development of IMRT, more accurate delineation of the tumor volumes and better dose distribution are obtained. The progression rate of early stage patients has largely reduced. Lee et al^23^ found the 5-year MFS could be reduced by 11%, which is benefit from the increased local control rate, by the IMRT group compared to the 2D-CRT group. Lai et al^14^ retrospectively analyzed 1276 patients who received radical 2D-CRT or IMRT and demonstrated improvement of local relapse-free survival (LRFS) in patients treated with IMRT than with 2D-CRT, especially in T1 disease (100% vs 94.4%, *P* = 0.016). It is reasonable to presume that the previously improved local/distant control by concurrent chemotherapy may probably be replaced by the modern radiotherapy technique (IMRT). However, there is still no randomized trial that directly compares IMRT alone with concurrent chemotherapy + IMRT. A retrospective analysis^15^ found concurrent chemotherapy + IMRT and IMRT alone did not differ in terms of 3-year local/regional control, MFS and OS. But there were only 8 patients in their CCRT group, probably inducing big bias. Furthermore, in Sun's study,^24^ concurrent chemotherapy even failed to improve survival rates (5-year MFS: 79.0% vs 80.8%, *P* = 0.998; PFS: 70.5% vs 68.8%, *P* = 0.480) but increased the severity of acute toxicities for patients with advanced locoregional disease when using the IMRT technique.

Our data first showed that omission of weekly-cisplatin chemotherapy resulted in comparable survival outcomes to CCRT in IMRT populations. OS, PFS, RFS, and MFS were excellent and did not differ between 2 treatment groups. Despite its retrospective profile, the study captured all T1-2N1 NPC patients treated with IMRT alone or cisplatin-CCRT between 2009 and 2011 in our center that would avoid the selection bias. And the population paired-based cohort would make the outcome more reliable. Our results were accordance to the previously reported outcomes by other centers. Patients achieved an extremely high complete response of 95.3% at 3 months after RT completion. The 3-year OS rate was remarkable, which reached 94.8% and 100% in the IMRT alone and CCRT group, respectively. Although weekly-DDP chemotherapy was tolerable and only increased mild to moderate toxicities (leukopenia and anemia), it did not improve the local/distant control and overall survival (*P* > 0.05). Late toxicities were similar in 2 groups to the last follow-up and with longer-term observations, the incidence and severity of late complications should approximate their true occurrence.

With the satisfactory results achieved by the modern radiation technique, no matter along with or without concurrent chemotherapy, we should reconsider the necessity of chemotherapy for early stage NPC when IMRT, instead of 2D-CRT, has been adopted. Even so, until more data from ongoing and future randomized trials are available on the survival and toxicity outcome, we must maintain a cautious and evidence-based approach to the management of stage II NPC patients.
